# Independent modulation of individual genomic component transcription and a cis-acting element related to high transcriptional activity in a multipartite DNA virus

**DOI:** 10.1186/s12864-019-5901-0

**Published:** 2019-07-11

**Authors:** Nai-Tong Yu, Hui-Min Xie, Yu-Liang Zhang, Jian-Hua Wang, Zhongguo Xiong, Zhi-Xin Liu

**Affiliations:** 1 Key Laboratory of Biology and Genetic Resources of Tropical Crops, Ministry of Agriculture and Rural Affairs, Institute of Tropical Bioscience and Biotechnology, Chinese Academy of Tropical Agricultural Sciences, Haikou, 571101 China; 2 School of Plant Sciences and BIO5 Institute, University of Arizona, Tucson, 85721 USA

**Keywords:** Multipartite DNA virus, *Banana bunchy top virus*, Transcription, Independent modulation, Cis-acting element

## Abstract

**Background:**

The genome of *Banana bunchy top virus* (BBTV) consists of at least six circular, single-stranded DNA components of ~ 1 kb in length. Some BBTV isolates may also carry satellite DNA molecules that are not essential for BBTV infection. The relation between multipartite DNA virus replication and their transcriptional levels and the underlying mechanism remain unclear.

**Results:**

To understand the coordinated replication and transcription of the multiple genomic components, the absolute amounts of each BBTV DNA component were measured by real-time PCR (qPCR), and their transcriptional levels were determined by RNAseq and reverse transcription-qPCR (qRT-PCR). Significant differences were found in the absolute amounts of individual BBTV genomic components. Transcriptional levels of each BBTV genomic component obtained from the RNAseq data matched closely to those obtained from qRT-PCR, but did not correspond to the absolute amount of each DNA component. The ratio of transcript over DNA copies ranged from 46.21 to 1059.44%, which was possibly regulated by the promoter region in the intergenic region of each component. To further determine this speculation, the promoter region of the DNA-S, −M or -N was constructed to the upstream of green fluorescent protein (GFP) gene for transient expression by agrobacterium-mediated transformation method. The qRT-PCR showed the highest transcriptional activity was promoted by DNA-N promoter, about 386.58% activity comparing with CaMV 35S promoter. Confocal microscopy observation showed that the intensity of green fluorescence was corresponding to that of qRT-PCR.

**Conclusions:**

Our data clearly showed that BBTV was able to control the transcriptional level of each DNA component independently by through the promoter sequences in the intergenic region. Moreover, a cis-acting element from DNA-N component had a high transcriptional activity.

**Electronic supplementary material:**

The online version of this article (10.1186/s12864-019-5901-0) contains supplementary material, which is available to authorized users.

## Background

*Banana bunchy top virus* (BBTV) causes a severe disease of banana worldwide, including Southern China [1–3]. BBTV is a member of the genus *Babuvirus* in the family *Nanoviridae.* Its genome contains at least six circular single-stranded DNA components of ~ 1 kb in size [4–10]. Some BBTV isolates may also carry 1 ~ 3 satellite DNA components that are not essential for BBTV infectivity [11, 12]. With the exception of DNA-U3, each BBTV genomic component encodes a single protein with known functions. DNA-R encodes a replication initiation protein (Rep) that introduces single-stranded DNA breaks and recirculates unit-length DNA genomes during replication [13, 14]. DNA-S encodes a capsid protein (CP) that packs each genome segment individually [15, 16]. DNA-M encodes a movement protein (MP) that allows cell-to-cell spread of the virus and functions also as a viral silencing suppressor [16, 17]. DNA-C encodes a cell-cycle link protein (Clink) that interferes with the cell cycle and promotes viral DNA synthesis in the host cells [18]. DNA-N encodes a nuclear shuttle protein (NSP) that translocates the virus out of nucleus [19]. The satellite molecules encode an assistant replication initiation protein (RepA) with a function similar to that of the Rep [11, 12].

BBTV and other similar viruses pack their multipartite genomic components separately in the virions, and must have all the essential components to co-infect host in order to constitute a functional virus [20, 21]. However, the significance of this biological process has not been completely understood yet. In *Faba bean necrotic stunt virus* (FBNSV), a member of the genus *Nanovirus*, the differential frequencies of nanoviral components affected the multipartite virus accumulation and symptom development, and the differential frequencies were host-specific [22]. Their data raised the possibility that multiple component viruses gain additional flexibility by regulating the copy number of individual genome components. This together with the differential transcriptional regulations of individual genomic components would offer additional plasticity in gene expression to allow multipartite DNA viruses to adapt to various conditions and maintain optimal fitness. The variations in the amount of BBTV individual DNA components and their transcript levels have not been reported to date. In this study, we conducted a comprehensive analysis of the amounts of individual DNA components and their transcript levels in BBTV-infected banana leaves to test this hypothesis.

Transcriptomic sequencing technology has been widely used as a tool to study the interaction between plant virus and host [23], as well as plant virus identification [24, 25]. Here, in order to understand the coordinated replication and transcription of the BBTV genomic components, transcriptomic sequencing analysis in combination with qPCR and qRT-PCR methods were used to measure the amounts of DNA and the RNA transcripts of the individual BBTV genomic components during the late stage of viral infection. To determine whether the transcriptional activity is regulated by the promoter region in the intergenic region of each component, the promoter sequences of DNA-S, −M or -N were studied by fusing with green fluorescent protein (GFP) gene. This study quantifies the DNA amount and RNA levels for each component of BBTV genome, and provides evidence to suggest independent modulation of transcriptional levels by the promoter region of each component in nanoviruses.

## Results

### RNAseq analysis revealed differential levels of RNA transcription of each BBTV component

The high-quality of total RNA samples from banana leaves (CONC ≥108 ng/μL; OD_260/280_ = 2.01 ~ 2.12; RIN ≥ 6.6) were obtained and conformed to the cDNA library preparation requirement. In total, 59.28, 51.46, 56.13 and 56.86 million raw reads were generated from B2, B4, H4 and H5 banana leaves respectively, and 56.22, 48.75, 53.34 and 52.61 million clean reads were further generated of these samples. The quality of Q20 percentage was over 96.37% for each sample (Table 1). The raw data of B2 B4, H4 and H5 is available in the NCBI database under the accession number SRP129855.

**Table 1:** Summary statistics of four transcriptome sequencing data and quality assessment

Sample	Raw reads	Clean reads	Clean bases	Error rate (%)	Q20 (%)	Q30 (%)	GC content (%)
B2_1	29,637,569	28,112,101	2.81G	0.04	97.13	91.48	50.36
B2_2	29,637,569	28,112,101	2.81G	0.04	96.42	90.35	50.40
B4_1	25,730,453	24,376,274	2.44G	0.04	97.10	91.37	51.08
B4_2	25,730,453	24,376,274	2.44G	0.04	96.44	90.35	51.12
H4_1	28,063,391	26,670,630	2.67G	0.04	97.00	91.19	50.88
H4_2	28,063,391	26,670,630	2.67G	0.04	96.50	90.48	50.92
H5_1	28,431,439	26,303,809	2.63G	0.04	97.09	91.38	50.68
H5_2	28,431,439	26,303,809	2.63G	0.04	96.37	90.26	50.73

Q20 (%): The percentage of sequences with a sequencing error rate lower than 1%

The mapped BBTV reads were initially assembled into contigs in the CodonCode Aligner 6.0.2 (CodonCode, Centerville, MA) and were further used to identify the possible BBTV genotypes. The result showed that there was apparently only one BBTV genotype in B2 sample, highly homologous to the BBTV Haikou isolate (FJ463042 ~ FJ463047). Additionally, it contained two satellite DNA components, designated as NewS2 and Sat4. Sequencing of three random clones of the amplified each BBTV component confirmed the assembled RNA sequences and filled the missing gaps in the assembled contigs. The assembled full-length nucleotide sequences of BBTV B2 isolate DNA-R (MG545610), DNA-U3 (MG545611), DNA-S (MG545612), DNA-M (MG545613), DNA-C (MG545614), DNA-N (MG545615), DNA-Sat4 (MG545616) and DNA-NewS2 (MG545617) were submitted into the GenBank. These sequences were used to remap the RNAseq reads to obtain more accurate, relative transcript levels for each BBTV component. According to the value of RPKM calculated from the mapping data, DNA-N (852,540.40), DNA-S (631,875.55) and DNA-U3 (564,461.46) components were highly expressed, followed by the DNA-M (111,881.29) with moderate expression. The DNA-R component (42,961.81) appeared to be expressed at a low level, but the DNA-C (3276.81) was expressed at the lowest, more than 250 fold less than that of DNA-N. In addition, both satellite DNA components expressed at low level and the satellite DNA of Sat4 has an even lower level (Table 2). The RPKM value of each BBTV component of B4 isolate was also calculated. The relative transcripts levels for each BBTV component were similar with the results that of B2 isolate. In details, DNA-N (983,805.86), DNA-S (583,410.23) and DNA-U3 (387,737.61) components were highly expressed, followed by the DNA-M (117,616.70) with moderate expression. The DNA-R component (12,539.84) expressed at a low level, but the DNA-C (3512.57) was expressed at the lowest, about 280 fold less than that of DNA-N. However, this BBTV genomic genotype contained only one satellite DNA component, Sat4, which expressed at a moderate level (Table 2).

**Table 2:** Reads coverage and RPKM for BBTV DNA components of B2 and B4 samples

Isolate	Component	GenBank no.	ORF (bp)	Read Counts	RPKM
B2	DNA-R	MG545610	104–964	1022	42,961.81
	DNA-U3	MG545611	143–409	4164	564,461.46
	DNA-S	MG545612	227–742	8956	631,875.55
	DNA-M	MG545613	282–632	1085	111,881.29
	DNA-C	MG545614	239–724	44	3276.81
	DNA-N	MG545615	277–741	10,953	852,540.40
	NewS2 (S2)	MG545617	62–919	1007	42,628.32
	Sat4	MG545616	49–903	398	16,789.22
B4	DNA-R	/	104–964	1208	12,539.84
	DNA-U3	/	143–409	11,583	387,737.61
	DNA-S	/	227–742	33,486	583,410.23
	DNA-M	/	282–632	4619	117,616.70
	DNA-C	/	239–724	191	3512.57
	DNA-N	/	277–741	51,184	983,805.86
	Sat4	/	49–903	9614	100,500.02

### Quantitative analysis by qPCR showed differential levels of BBTV genomic components.

**Fig. 1:**
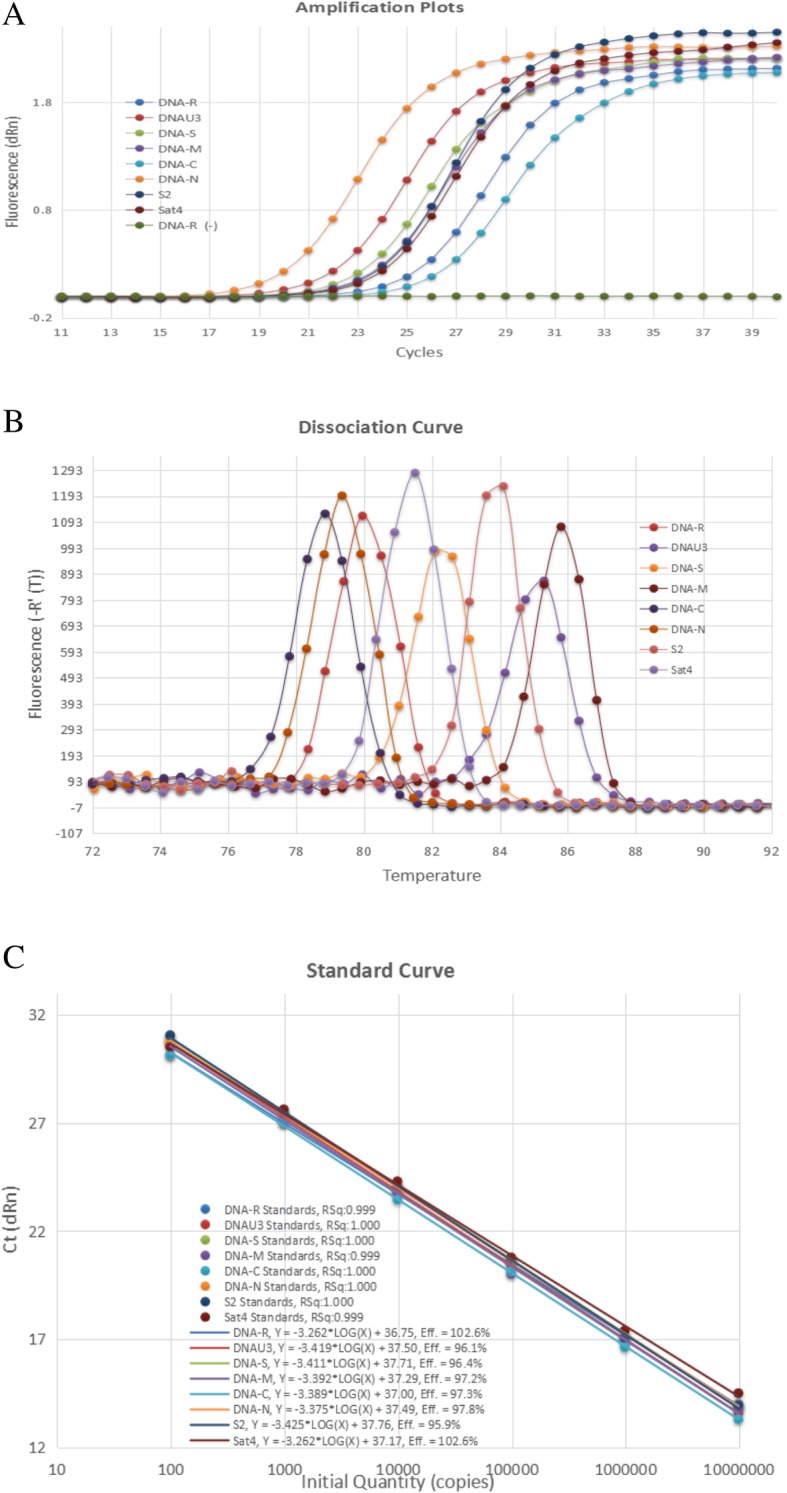
Plots of qPCR standard curves for each BBTV component**. a** The qPCR amplification curve of each component of BBTV genome. The X axis represents the number of cycles, while the Y axis represents the dRn value of fluorescence. **b** The melt curve of each component of BBTV genome. The X axis represents the temperature value, while Y axis represents the -R’(T) value of fluorescence. **c** The standard curves of DNA-R, DNA-U3, DNA-S, DNA-M, DNA-C, DNA-N, S2 and Sat4 were plotted. The X axis represents the number of DNA copies, while the Y axis represents the Ct value

The standard curves of each BBTV DNA component were constructed and the results showed that the optimized qPCR system with the specific primer pairs amplified BBTV genomic components highly efficiently (efficiencies of 95.5 to 102.6%) with correlation coefficients between 0.999 to 1.000 (Fig. 1).

**Fig. 2:**
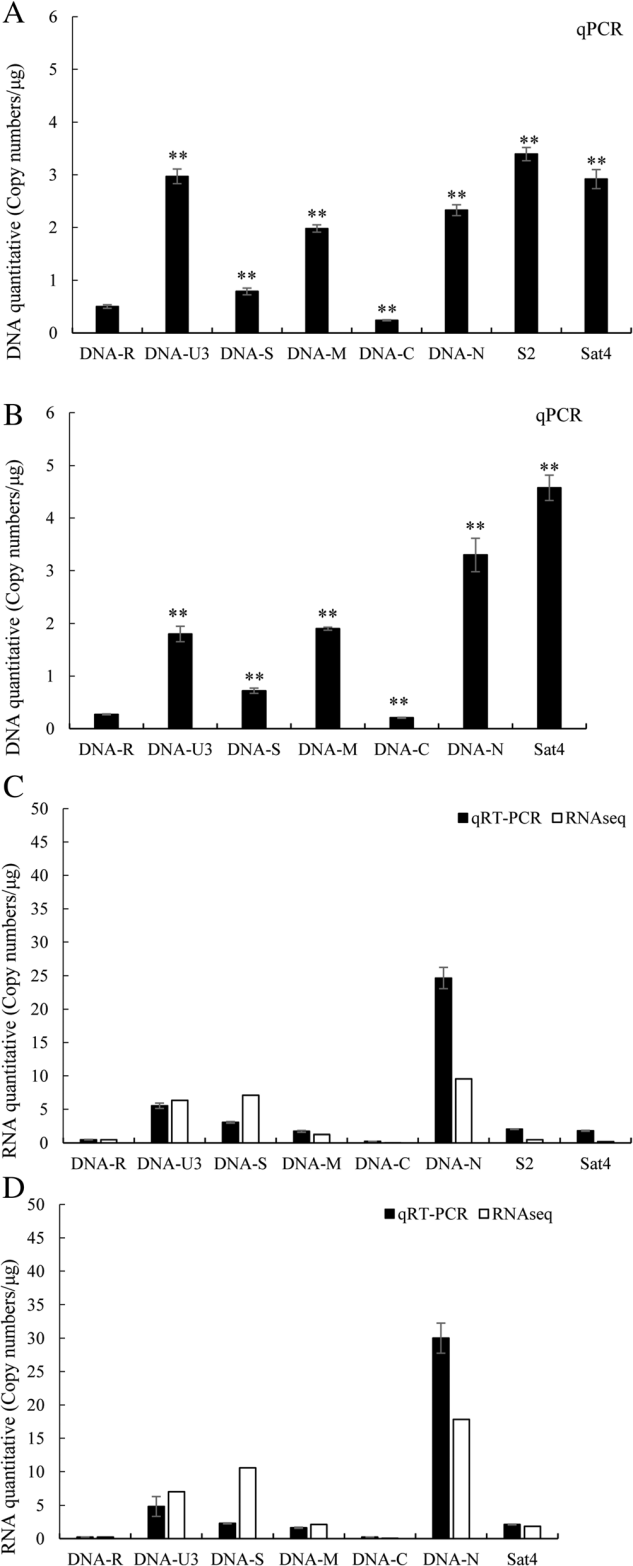
The quantitative analysis of DNA level and RNA level of BBTV genomic components**.** DNA content of BBTV each genomic components comparing with DNA-R from qPCR in B2 (**a**) and B4 (**b**) samples. The X axis represents the name of component, while Y axis represents the copy numbers. RNA content of BBTV each genomic components comparing with DNA-R from qRT-PCR and RNAseq in B2 (**c**) and B4 (**d**) samples. The X axis represents the name of component, while Y axis represents the copy numbers. P<0.01 was considered as extremely significantly different

The qPCR analysis of the BBTV DNA in virus-infected banana leaves showed that DNA-U3, DNA-N, and DNA-M components were represented in significantly higher copies than other essential DNA components in B2 sample. The DNA copy numbers of DNA-U3, DNA-N, and DNA-M were 2.97 copies/μg, 2.33 copies/μg, and 1.98 copies/μg, respectively. DNA-C, with the DNA copy number of 0.24 copies/μg, was 14 times lower than that of DNA-U3, the most abundant BBTV DNA component. Another two important essential components, DNA-R and DNA-S, were also present at lower levels of 0.50 copies/μg and 0.79 copies/μg, respectively. Interestingly, two satellite DNA components were also present at a high level, consistent with their parasitic nature. The copy numbers of S2 at 3.4 copies/μg and Sat4 at 2.92 copies/μg were as high as or higher than the most abundant BBTV component, DNA-U3 (Table 3 and Fig. 2a). In B4 sample, the most abundant of DNA-N, was the highest level of 3.3 copies/μg. DNA-U3 and DNA-M were also present at high levels of 1.80 copies/μg and 1.90 copies/μg, respectively. However, DNA-R and DNA-C were present at low levels of 0.27 copies/μg and 0.21 copies/μg, respectively. The satellite DNA component was also present at a high level, with the copy numbers of Sat4 at 4.57 copies/μg (Table 3 and Fig. 2b).

**Table 3:** Absolute quantitative analysis and their expression of the BBTV genomic components of B2 and B4 samples

Isolate	Component	DNA Ct(dRn)			DNA Aver. (copies/μg)	RNA Ct(dRn)			RNA Aver. (copies/μg)	RNA/DNA ratios (%)
		1	2	3		1	2	3		
B2	DNA-R	23.93	23.99	24.12	0.50	26.14	26.05	25.92	0.48	95.92
	DNA-U3	21.44	21.51	21.58	2.97	22.71	22.71	22.53	5.52	185.79
	DNA-S	23.61	23.85	23.71	0.79	23.78	23.84	23.72	3.03	384.35
	DNA-M	22.08	22.01	21.98	1.98	24.32	24.41	24.17	1.69	85.45
	DNA-C	24.91	24.8	24.82	0.24	27.05	27.07	26.92	0.22	91.62
	DNA-N	22.09	21.99	22.11	2.33	20.7	20.68	20.53	24.67	1059.44
	S2	21.54	21.49	21.6	3.40	24.36	24.42	24.37	2.01	59.26
	Sat4	21.99	21.99	21.84	2.92	24.65	24.64	24.54	1.77	60.71
B4	DNA-R	24.83	24.91	24.92	0.27	27.23	27.16	26.93	0.23	83.78
	DNA-U3	22.13	22.27	22.37	1.80	23.55	23.67	23.50	4.81	267.49
	DNA-S	23.77	23.97	23.83	0.72	24.15	24.23	24.23	2.28	316.14
	DNA-M	22.07	22.11	22.07	1.90	24.42	24.37	24.27	1.63	85.74
	DNA-C	25.01	25.07	25.13	0.21	27.17	27.11	26.97	0.21	101.96
	DNA-N	21.57	21.69	21.41	3.30	20.47	20.33	20.25	30.01	910.09
	Sat4	21.39	21.27	21.25	4.57	24.33	24.41	24.34	2.11	46.21

### Quantitative analysis by qRT-PCR confirmed differential levels of BBTV transcripts

To verify the differential transcript levels of each BBTV genomic component in B2 sample, qRT-PCR using the same qPCR primer pairs was deployed to quantitate BBTV transcripts. The most abundant mRNA copies were transcribed from DNA-N (24.67 copies/μg), followed by DNA-U3 (5.52 copies/μg). The least abundant mRNA copies were made from DNA-R (0.48 copies/μg) and DNA-C (0.22 copies/μg). The mRNA copies from other BBTV components ranged from 1.69 to 3.03 copies/μg. In B4 sample, the high abundant mRNA copies were transcribed from DNA-N (30.01 copies/μg), DNA-U3 (4.81 copies/μg) and DNA-S (2.28 copies/μg), respectively. The low abundant mRNA copies were transcribed from DNA-R (0.23 copies/μg) and DNA-C (0.21 copies/μg). The mRNA copies of satellite DNA components were present at moderate levels in both B2 and B4 samples (Table 3).

Although the absolute number of qRT-PCR quantification does not match the read numbers mapped to each gene in RNAseq, the relative proportions of the transcripts from each BBTV DNA component are closely matched from these two independent measurements (Fig. 2c and d). These data together confirmed the large disparities in the copy number of transcripts from each BBTV component. The most abundantly expressed DNA-N made 143-fold more mRNA than DNA-C did in B4 sample. Interestingly, DNA-R, encoding the master Rep, was expressed at a very low level, but the Rep genes from the two satellite DNAs were expressed at a moderate level, approximately 4 ~ 9-fold higher than DNA-R. DNA-S, a structural protein for virus assembly, and DNA-M, a movement protein, both were expressed at a moderate level (Fig. 2c and d).

### Independent modulation of transcript levels in each BBTV genomic component

**Fig. 3:**
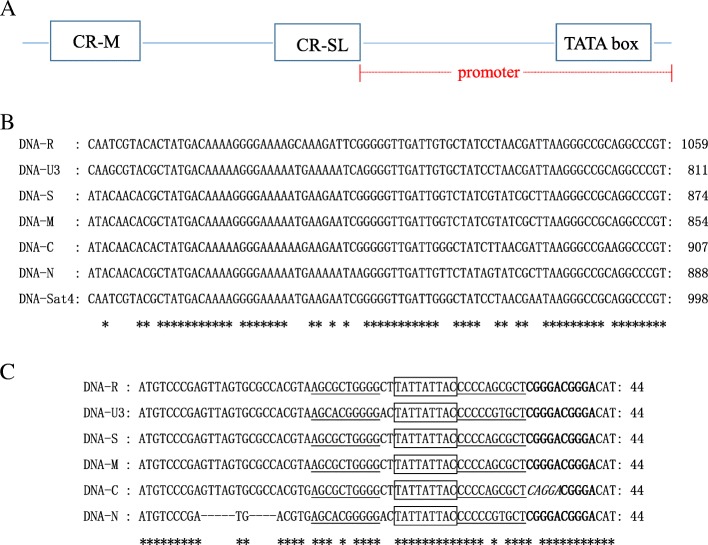
The diagram and sequence analysis of intergenic regions of BBTV each component. **a** The diagram of intergenic regions of BBTV each component; **b** Sequence aligned of major common regions (CR-M) of BBTV each component; **c** Sequence aligned of stem-loop common regions (CR-SL) of BBTV each component. TATTATTAC: Step-loop of highly conserved nine nucleotide sequences; ‘CGGGA’: Five nt repeat sequences; Reverse complement sequences between AGCGCTGGGG and CCCCAGCGCT or between AGCACGGGGG and CCCCCGTGCT; and the mutated ‘*CAGGA*’ sequences from ‘CGGGA’

The DNA copy and transcript level data clearly showed discordance. The most abundant DNA component did not produce the highest level of RNA transcripts. The ratio of transcript copies over DNA copies were calculated to measure transcriptional activities of each DNA component. These ratios ranged from 46.21 to 1059.44% (Table 3). BBTV components encoding catalytic proteins or regulatory proteins or movement proteins were transcribed at a relative lower level with a RNA/DNA ratio of 101.96% or less (Table 3). Sequences analysis revealed that the intergenic regions of six necessary components of the two isolates have typical replication and transcription regulatory regions in nanovirus (Fig. 3a), containing a conserved major common region (CR-M) and a conserved stem loop common region (CR-SL) (Fig. 3b and c). Further analysis indicated the nucleotide sequences of CR-M, CR-SL and the regions between the CR-M and CR-SL are high conserved between DNA-S and DNA-M, about 99% sequence identity, but the RNA/DNA ratios are large disparities. Therefore, we speculate that the promoter sequence in the intergenic region of each component is response to the transcriptional activity. Meanwhile, a TATA box was found in the promoter region of six necessary components, which is the binding sites of the TATA-binding protein (TBP) and transcription factors. In addition, the most actively transcribed BBTV component was DNA-N, which encodes a nuclear shuttle protein. About over ten copies of RNA transcripts per DNA copy were made from DNA-N (Table 3).

### A cis-acting element from DNA-N component had a high transcriptional activity

**Fig. 4:**
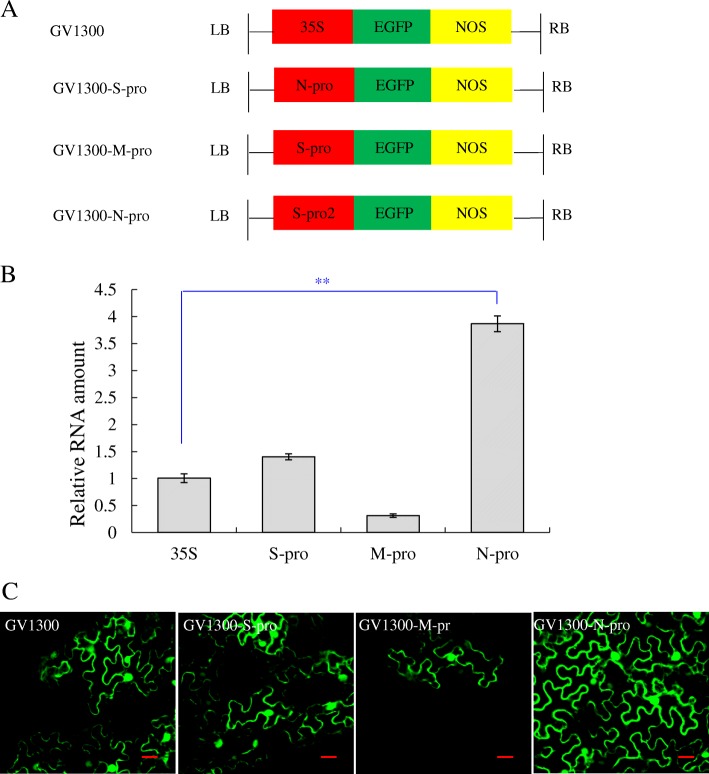
The transcriptional activity of DNA-S, −M and -N promoters by fusing with *GFP*. **a** The diagram of recombinant plasmids construction. GV1300, GV1300-S-pro, GV1300-M-pro, GV1300-N-pro. **b** The relative RNA amount of *GFP* transcripts transcribed by the different promoter sequences of DNA-S, −M and -N, respectively. P<0.01 was considered as extremely significantly different. **c** The green fluorescence was observed by a confocal microscopy. Scale bar, 20 μm

The transcriptional activity regulated by promoter region was analyzed by qRT-PCR, and the results indicated all *GFP* transcripts are detected at 72 h post transfection (hpt). Comparing with CaMV 35S promoter, the highest transcriptional level was promoted by DNA-N promoter (N-pro), about 386.58% transcriptional activity, followed by 140.23% of DNA-S promoter (S-pro). The transcriptional activity of DNA-M promoter (M-pro) was present at a low level, about 31.18% transcriptional activity (Fig. 4b). Meanwhile, confocal microscopy observation showed that the intensity of green fluorescence signal was corresponding to that of qRT-PCR (Fig. 4c). Therefore, BBTV individual genomic gene transcription was regulated by the promoter sequence in the intergenic region of each component, and a cis-acting element from DNA-N component had a high transcriptional activity for gene transcription.

## Discussion

BBTV is a multi-component nanovirus whose genome is composed of at least six circular ssDNA. Our data showed that the replication and transcription of each genomic component are independently modulated. The absolute quantitative analysis of BBTV components showed significant differences in the DNA and RNA contents. Transcripts levels determined by qRT-PCR, in accordance to the RNAseq data, did not correspond to the absolute amount of each DNA component. The most abundant transcripts came from DNA-N, followed by DNA-U3, and DNA-S, and the least abundant transcripts came from DNA-C, 143-fold less than those from DNA-N. The most abundant transcripts of DNA-N encoded a nuclear shuttle protein that moves BBTV DNA in and out nuclei [19, 26]. This abundant protein may have additional functions as it contains a ‘FNGSF’-motif which is capable of binding to banana Ras-GAP SH3-domain-binding protein (G3BP) and inhibiting the formation of stress granules (SGs) in host [27]. DNA-S, encoding a capsid protein (CP) that packages each genome segment individually, is expected to have a relative abundant level of transcripts. DNA-C encodes a Clink protein at the early stage of viral infection, regulating the host cell into S phase, which facilitate the viral DNA replication [19, 28]. Northern blot showed that the mRNA content of the DNA-C component was much less than that of the other components, which was consistent with the results of this study [26]. Similar with *Potexvirus* satellite RNA and *Geminivirus* betasatellite DNA [29, 30], BBTV from B2 isolate had two satellite components, but B4 isolate carried only one satellite component. These satellite molecules are not essential for infectivity. Satellite molecules, the lowest ratio of RNA/DNA, encode assistant replication initiation protein (RepA) with a function similar to that of the Rep, but it only introduces single-stranded DNA breaks and recirculates unit-length DNA of self-components during the replication in host [12].

Regulations at the gene replication, transcriptional, and translational levels in the biological process have been well characterized. However, the gene copy numbers are now also known to play important roles. The multiple copies of genes within a single cell would greatly promote gene expressions in most organisms [31, 32]. For example, gene copy number variations can be found in genetic diseases, and in protozoa and bacteria that response to stress [33, 34]. In *Faba bean necrotic stunt virus* (FBNSV), different amounts of eight genomic components were found to affect virus accumulation and the symptoms formation on the host plant [22]. In this report, we also showed a member of *Babuvirus*, BBTV, exhibited copy variations of viral genomic components. Comparing with the DNA variations of genomic components in FBNSV, a member of *Nanovirus*, BBTV has the similar trends in the study. The DNA amount of DNA-C, DNA-R and DNA-S components is lower than those of DNA-M, DNA-N and DNA-U3 components, which is consistent with that of FBNSV. These data suggest that the copy number variation of viral genomic components perhaps is a common mechanism for single-stranded, multipartite DNA viruses to regulate their gene expression.

The replication of viruses takes place in a complex environment, which the virus and the host continuously compete for resources. For a multipartite virus, at least one copy of each segment is required to infect the host. The significant variations in the amounts of each BBTV genomic component may imply an unforeseen benefit for multipartite viruses. For example, the minimum copy number and transcription level of the DNA-C component, which encodes Clink protein to interfere the host cell cycle, are sufficient enough to change the cell cycle for the virus benefit but not too high to upset normal plant development [35, 36]. The low copy number and transcript level of DNA-R, which encode a replication initiation protein, are also sufficient enough to maintain enzymatic activities for replication initiation and progeny ssDNA circularization but not in excess. Similarly the selfish satellite DNA components, S2 and Sat4, tune down their transcription levels although they have very high DNA copy numbers. Interestingly, DNA-N component, the highest transcription level among genomic components, has multiple functions during the virus infection. With the exception of nuclei transportation and stress response, the protein interacted and re-distributed the BBTV CP in the plant cell [37]. The multiple functions of DNA-N encoding protein require its high transcription activity. On the other hand, some variations are difficult to explain with the current understanding. For example, the high copy number and transcripts of DNA-U3, which may or may not encode a very small protein of unknown function, remain a mystery. The mechanism regulating differential DNA content via specific viral sequences should be studied further.

## Conclusions

Our data clearly showed that BBTV was able to control the transcriptional level of each DNA component independently. Although the sizes of each BBTV DNA component are similar, the transcriptional level of the component could be different by more than of a hundred folds, with some genomic component highly transcribed and some poorly transcribed. Further experiments indicated the differently transcriptional level is regulated by the promoter sequence in the intergenic region of each component. Moreover, a cis-acting element from DNA-N component had a high transcriptional activity was obtained. Together, our data provided compelling evidence of independent modulation of transcriptional activities for each BBTV genomic component.

## Materials and methods

### Plant materials

Samples of BBTV-infected (B2 and B4) and healthy (H4 and H5) banana leaves were collected from Haikou, Hainan, China in October, 2013. The banana, the main planting line in Hainan province, is the species of *Musa* AAA Cavendish subgroup cv. Brazil. The diseased banana plants were displaying symptoms of bunchy top and narrow leaf in the late stage of BBTV infection, while no symptoms were observed on the healthy banana samples. Four samples were kept at − 80 °C in the laboratory.

### RNA preparation and qualification

Total RNAs of banana leaves were extracted by using TRIzol® reagent according to the manufacturer’s instruction (Thermo Fisher Scientific, Waltham, MA, USA), and further treated with DNase I (Takara, Beijing) to remove DNA contamination. The RNA integrity (RIN) was assessed using the RNA Nano 6000 Assay Kit of the Agilent Bioanalyzer 2100 system (Agilent Technologies, CA, USA). RNA purity was checked using the NanoPhotometer® spectrophotometer (IMPLEN, CA, USA), and RNA concentration was measured using Qubit® RNA Assay Kit in Qubit® 2.0 Flurometer (Life Technologies, CA, USA).

### Library preparation for transcriptome sequencing

A total amount of three μg RNA per sample was used as input materials for the cDNA libraries construction. Sequencing libraries were generated using NEBNext® Ultra™ RNA Library Prep Kit for Illumina® (NEB, USA) following manufacturer’s recommendations and index codes were added to differentiate sequences from each sample. The detailed steps were described in the references [38, 39]. First strand cDNA was synthesized by using random hexamer primer and M-MuLV reverse transcriptase with no RNase H activities. Second strand cDNA synthesis was subsequently performed using DNA polymerase I and RNase H. Clustering and sequencing were performed by the Experimental Department at Novogene (Beijing, China). Briefly, the clustering of the index-coded samples was conducted on a cBot Cluster Generation System using TruSeq PE Cluster Kit v3-cBot-HS (Illumia) according to the manufacturer’s instructions. After cluster generation, the prepared libraries were sequenced on an Illumina Hiseq 2000 platform and 100-bp paired-end reads were generated.

### Data analysis of transcriptome sequencing

Raw reads in the fastq format were firstly cleaned to remove low quality reads and reads containing adapters and ploy-Ns. Q20, Q30 and GC content of the clean data were calculated to evaluate the overall quality of the clean reads. All the downstream analyses were based on the clean data with high quality.

### Reads mapping and gene expression level analysis of the BBTV genomes

The reference BBTV genomes and gene model annotation files were downloaded from NCBI website (https://www.ncbi.nlm.nih.gov/nuccore/?term=banana%20bunchy%20top%20virus) directly. Index of the reference genome was built using Bowtie v2.0.6 and paired-end clean reads were aligned to the reference genome using TopHat v2.0.9 [40]. HTSeq v0.5.4p3 was used to count the number of reads mapped to each component of the BBTV genome [41]. Meanwhile, RPKM of each gene of BBTV genome was calculated based on the length of the gene and reads count mapped to this gene. RPKM, Reads Per Kilobase of exon model per Million mapped reads, considers the effect of sequencing depth and gene length for the reads count at the same time, and is now the most commonly used method for estimating gene expression levels [42].

### Primers design and optimization of qPCR

The large disparities in the transcript levels of individual BBTV components in the RNAseq analysis prompted us to exam the levels of individual BBTV genomic components and their transcript levels in the virus infected leaves by independent qPCR assays. Primer pairs for qPCR amplification of each BBTV component (Table 4) were designed as previously described [4–6]. Total DNAs of B2, B4, H4 and H5 banana leaves were extracted by the CTAB method [43]. To measure accurately the copy numbers of the BBTV genomic components and their transcripts, we first assessed the specificity of the qPCR primer pairs. Regular PCR was set up by using the *EasyTaq* DNA Polymerase kit (TransGen, Beijing, China) and amplified fragments of BBTV genomic components, about 150 bp in sizes, were cloned into pMD19-T. Three randomly selected positives clones of BBTV each component were sequenced. The sequencing results confirmed the specificity of the qPCR primers.

**Table 4:**
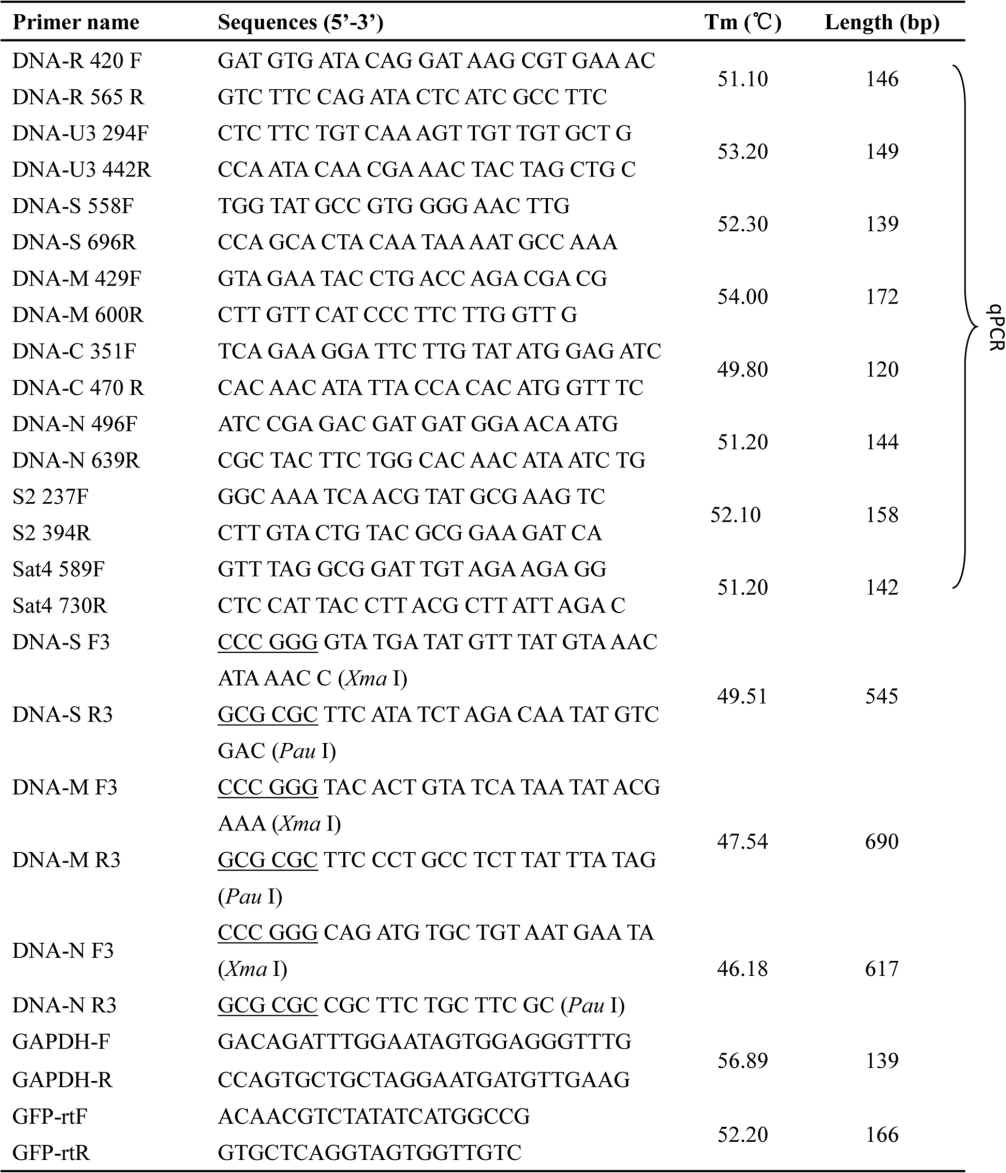
Primers used in the study

We further optimized the qPCR reaction system by testing various annealing temperatures and primers ratios by using SYBR Premix Ex Taq (TaKaRa, China) on the Stratagene Mx3005 machine. The specific target bands of each component were amplified from different annealing temperatures ranging from 47 °C to 61 °C (Additional file 4: Figure S1). Except DNA-R and DNA-S, the brightness of the amplified fragments from DNA-U3, DNA-M, DNA-C, DNA-N, S2 and Sat4 components decreased with the increase of temperature, indicating that the amplification efficiency decreased with the increase of temperature. Further analysis indicated that the amplification efficiency did not change largely at the annealing temperatures of 47 °C, 49 °C, 51 °C or 53 °C. In the study, the annealing temperature of 53 °C was selected as the annealing temperature of the qPCR in order to ensure both the specificity and the efficiency of the qPCR systems.

Based on the optimized melting temperature, the concentrations and ratios of forward and reverse primers were also tested at the ratio of 0.2/0.2, 0.2/0.3, 0.2/0.4, 0.3/0.2, 0.3/0.3, 0.3/0.4, 0.4/0.2, 0.4/0.3, and 0.4/0.4 μM. The Ct values from different concentrations and ratios of forward and reverse primers ranged from 14 to 21 (Additional file 1: Table S1). The highest Ct values were obtained when the concentrations of the forward and reverse primers were both at 0.2 μmol/L while the lowest Ct values were found when the concentrations of the forward and reverse primers were both at 0.4 μmol/L. For subsequent experiments, the primers concentrations of both forward and reverse were chosen at 0.4 μmol/L.

Based on the above optimization results, the qPCR reaction system and conditions were determined as follows: 25 μL reaction system containing 12.5 μL of 2 × SYBR Premix Ex Taq, 1 μL of 10 μM qPCR primers F/R, 2.0 μL of Template DNA, 0.5 μL of 50 × ROX Reference Dye II, 9.0 μL of ddH_2_O. The program of qPCR was follows: 95 °C 3 min; 40 cycles of 95 °C 30 s, 53 °C 20 s, 72 °C 20 s; fluorescence signal was collected each cycle after 53 °C 20 s.

### Cloning of full-length BBTV genomic DNA

Full-length DNA of the eight BBTV genomic components were amplified with the full-length specific primers (Additional file 2: Table S2), and cloned into the pMD19-T vector (Takara, Beijing) by TA-cloning. PCR amplification was performed as described above, but with the extension step increased to one minute. The recombinant plasmids were identified by PCR with the M13 forward and reverse primers and further confirmed by sequencing. The DNA concentration of each recombinant plasmid was measured by a nucleic acid analyzer (NanoDrop 2000, Thermo Fisher Scientific), and the plasmids were used to establish standard curves for qPCR. The concentrations of these recombinant plasmids varied from 185.4 to 531.8 ng/μL (Additional file 3: Table S3).

### Quantitative analysis of BBTV DNA components and their expression levels

To measure the copy number of BBTV genomic DNA in virus infected leaves, standard curves of each BBTV DNA component were constructed. The full-length fragments of BBTV genomic components were amplified and cloned into pMD19-T above. The copy number of each BBTV component plasmid was calculated by the following formula: Copy number/μL = [Concentration_plasmid_ (ng/μL) × 1 μL] × 10^− 9^ / [(Length_plasmid_ + Length_insert_) × 660] × 6.02 × 10^23^. Each recombinant plasmid was diluted to 9.83 × 10^6^ copies/μL as an initial template. The initial template was further diluted to 10^− 1^, 10^− 2^, 10^− 3^, 10^− 4^ and 10^− 5^ by ddH_2_O, and were used for establishing the standard curves with the optimized qPCR condition in the Stratagene Mx3005 machine. The 1000 times diluted total DNA from healthy banana leaves was used as a negative control.

The viral DNA copy numbers of each component of BBTV in per μg of banana leaf was measured three times independently by qPCR with the optimization conditions using the total DNA extracted from BBTV-infected and healthy banana leaves as template. The viral RNA copy numbers transcribed from each component of BBTV in per μg of banana leaf was also measured three times independently with qPCR using the first stranded cDNAs as templates and the same qPCR primers pairs. First-strand cDNA was synthesized from 2.0 μL of total RNA using 1.0 μL of random hexamer primer (10 μM) and 0.5 μL M-MLV reverse transcriptase (200 U/μL, Promega, Madison, WI, USA) at 42 °C for 30 min. The qPCR amplification curves of BBTV components were generated and the absolute quantitative was obtained for each BBTV genomic component. Student’s t-test was used to evaluate the differences.

In order to quantify the relation between the read numbers and qRT-PCR, the copy numbers of the DNA-R reads counts (RPKM) were conversion to the absolute number according to the absolute number of DNA-R in qRT-PCR quantification. Then, the copy numbers of other components reads counts were further converted with the relative proportions to DNA-R reads counts.

### The transcriptional activity of DNA-S, −M and -N promoters by fusing with *GFP*

To further determine the transcriptional activity regulated by promoter sequence of each component, the promoter sequences of DNA-S (+ 45 ~ + 226), −M (+ 45 ~ + 281) and -N (+ 45 ~ + 276) were amplified by pair primers (Table 4) and constructed into the upstream of *GFP* in GV1300 vector (Fig. 4a). The recombinant plasmids, GV1300, GV1300-S-pro, GV1300-M-pro and GV1300-N-pro, were separately transformed into the *Agrobacterium tumefaciens* GV3101 competent cells by freeze-thaw method and positive clones were subsequently identified by PCR. Then, a rapid, transient expression method of fluorescent fusion proteins in tobacco plants was conducted according to the references [44]. At 72 hpt, total RNA was extracted and reverse transcribed into the first-strand cDNA as described above. To detect the effect of different promoters on gene transcription, the relative RNA amounts of *GFP* were measured by quantitative real-time PCR (qRT-PCR) using SYBR Premix Ex Taq (TaKaRa, China). Three biological replicates were assayed. The internal control, *GAPDH*, was used to normalize each sample and *GFP* transcription level was evaluated based on 2^-△△Ct^ method. Student’s t-test was used to evaluate the differences. Meanwhile, the intensity of green fluorescence was observed by a confocal microscopy (FluoView FV1000D IX81; Olympus, Tokyo, Japan) observation under 488 nm and 546 nm.

## Additional files

Additional file 1: **Table S1.** The Ct value of BBTV each component by different concentrations of forward and reverse primers. (DOCX 16 kb)

Additional file 2: **Table S2.** Primers designed for BBTV genomic components. (DOCX 15 kb)

Additional file 3: **Table S3.** The DNA concentration of recombinant plasmids of BBTV genomic components. (DOCX 16 kb)

Additional file 4: **Figure S1.** Optimization of annealing temperatures for each component of BBTV. (PDF 99 kb)

## Data Availability

The raw data of B2, B4, H4 and H5 is available in the NCBI database under the accession number SRP129855. The genome of BBTV isolate B2 is available in the NCBI database under the accession numbers of DNA-R (MG545610), DNA-U3 (MG545611), DNA-S (MG545612), DNA-M (MG545613), DNA-C (MG545614), DNA-N (MG545615), DNA-Sat4 (MG545616) and DNA-NewS2 (MG545617), respectively.

## Abbreviations

BBTV: *Banana bunchy top virus*
Clink: Cell-cycle link protein
CP: Capsid protein
CR-M: Major common region
CR-SL: Stem loop common region
FBNSV: *Faba bean necrotic stunt virus*
G3BP: Ras-GAP SH3-domain-binding protein
MP: Movement protein
NSP: Nuclear shuttle protein
qPCR: Real-time quantitative PCR
qRT-PCR: Reverse transcription-qPCR
Rep: Replication initiation protein
RepA: Assistant replication initiation protein
SGs: Stress granules
