# Supplementary material for: Independent modulation of individual genomic component transcription and a cis-acting element related to high transcriptional activity in a multipartite DNA virus
Source: BMC Genomics. 2019 Jul 11;20:573. doi: 10.1186/s12864-019-5901-0 (PMC6625112; doi:10.1186/s12864-019-5901-0)
Supplement: Supplementary file 1 — Table S1. The Ct value of BBTV each component by different concentrations of forward and reverse primers. (DOCX 16 kb) [file 12864_2019_5901_MOESM1_ESM.docx]

**Table S1.** The Ct value of BBTV each component by different concentrations of forward and reverse primers.

| **Ct value of each component** | **The concentration of primers F/R (μmol/L)** | | | | | | | | |
| --- | --- | --- | --- | --- | --- | --- | --- | --- | --- |
|  | 0.2/0.2 | 0.2/0.3 | 0.2/0.4 | 0.3/0.2 | 0.3/0.3 | 0.3/0.4 | 0.4/0.2 | 0.4/0.3 | 0.4/0.4 |
| DNA-R (Ct) | 20.50 | 19.87 | 18.90 | 19.67 | 18.75 | 18.03 | 20.01 | 19.34 | 17.24 |
| DNA-U3 (Ct) | 17.38 | 16.27 | 15.42 | 16.69 | 16.81 | 15.40 | 17.20 | 16.64 | 14.59 |
| DNA- S (Ct) | 20.65 | 20.23 | 19.34 | 20.45 | 19.78 | 19.12 | 20.43 | 19.87 | 17.43 |
| DNA-M (Ct) | 18.68 | 18.45 | 17.63 | 18.06 | 17.57 | 16.48 | 18.67 | 17.98 | 15.55 |
| DNA-C (Ct) | 21.33 | 21.05 | 20.05 | 21.44 | 20.44 | 19.21 | 21.21 | 20.53 | 18.47 |
| DNA-N (Ct) | 18.87 | 18.54 | 17.90 | 18.43 | 17.45 | 17.98 | 18.88 | 17.68 | 15.68 |
| S2 (Ct) | 17.57 | 16.52 | 15.68 | 16.90 | 17.00 | 15.68 | 17.43 | 16.90 | 14.75 |
| Sat4 (Ct) | 19.11 | 18.80 | 18.09 | 19.26 | 17.80 | 17.69 | 19.12 | 18.02 | 15.91 |
