# Supplementary material for: Independent modulation of individual genomic component transcription and a cis-acting element related to high transcriptional activity in a multipartite DNA virus
Source: BMC Genomics. 2019 Jul 11;20:573. doi: 10.1186/s12864-019-5901-0 (PMC6625112; doi:10.1186/s12864-019-5901-0)
Supplement: Supplementary file 2 — Table S2. Primers designed for BBTV genomic components. (DOCX 15 kb) [file 12864_2019_5901_MOESM2_ESM.docx]

**Table S2.** Primers designed for BBTV genomic components

| **Primer name** | **Sequences (5’-3’)** | **Tm (℃)** | **Template** |
| --- | --- | --- | --- |
| DNA-R R158 | TCC AGT GAT GCG GGA TGA GTT | 51.8 | FJ463042.1 |
| DNA-R F159 | GAA GTG AAG GCG GAT TGT TGA TG |  |  |
| DNA-U3 R841 | CTT AGC CAC GAA GGA AGG AAT CT | 46 | FJ463043.1 |
| DNA-U3 F841 | GCA ATC AAA TCT AAC CGT TCA TCC |  |  |
| DNA-S R995 | CGG ATA AGG ATG AGA ACC ACC | 46.6 | FJ463044.1 |
| DNA-S F995 | GGC ACA ACT ACA CCT TCC TTC TC |  |  |
| DNA-M R319 | AAT GGT TTC TGT TAG TTG GAG CA | 46 | FJ463045.1 |
| DNA-M F320 | TTC AAA GAA TAG TTT CAC CCG C |  |  |
| DNA-C R563 | CAA GAG TTG TAT TGT GAT GAG G | 55 | FJ463046.1 |
| DNA-C F563 | GAT ATA CCG AGT AGT CAC CAC C |  |  |
| DNA-N R266 | AAG CAG AAG CGA TGG ATT GGG | 50.8 | FJ463047.1 |
| DNA-N F269 | GCT TCG CAT ACG CTC TGA TTT A |  |  |
| S2 R985 | ACG CTA TGC CGT ACA ACC AAG TC | 45.2 | AF216222.1 |
| S2 F987 | CGT GTT ATC GTA GTG GTG GGG TCC |  |  |
| Sat4 R302 | CGT ACT GTT CTA ATG AAG CCC TA | 47.9 | EU430730.1 |
| Sat4 F304 | ACG CCT TGT TGT CGT AAT CTG |  |  |
