# Supplementary material for: Independent modulation of individual genomic component transcription and a cis-acting element related to high transcriptional activity in a multipartite DNA virus
Source: BMC Genomics. 2019 Jul 11;20:573. doi: 10.1186/s12864-019-5901-0 (PMC6625112; doi:10.1186/s12864-019-5901-0)
Supplement: Supplementary file 3 — Table S3. The DNA concentration of recombinant plasmids of BBTV genomic components. (DOCX 16 kb) [file 12864_2019_5901_MOESM3_ESM.docx]

**Table S3.** The DNA concentration of recombinant plasmids of BBTV genomic components

| **Constructed plasmid** | **Concentration (ng/μL)** | **OD_260_/OD_280_** | **OD_260_/OD_230_** |
| --- | --- | --- | --- |
| pMD19T- BBTV- DNA- R | 248.4 | 1.87 | 2.10 |
| pMD19T- BBTV- DNA-U3 | 212.9 | 1.86 | 2.15 |
| pMD19T- BBTV- DNA-S | 531.8 | 1.87 | 2.22 |
| pMD19T- BBTV- DNA-M | 370.6 | 1.88 | 2.04 |
| pMD19T- BBTV- DNA-C | 523.5 | 1.87 | 2.23 |
| pMD19T- BBTV- DNA-N | 185.4 | 1.87 | 2.13 |
| pMD19T- BBTV- S2 | 290.6 | 1.87 | 2.24 |
| pMD19T- BBTV- Sat4 | 440.1 | 1.86 | 2.20 |
