# Supplementary material for: Independent modulation of individual genomic component transcription and a cis-acting element related to high transcriptional activity in a multipartite DNA virus
Source: BMC Genomics. 2019 Jul 11;20:573. doi: 10.1186/s12864-019-5901-0 (PMC6625112; doi:10.1186/s12864-019-5901-0)
Supplement: Supplementary file 4 — Figure S1. Optimization of annealing temperatures for each component of BBTV. (PDF 99 kb) [file 12864_2019_5901_MOESM4_ESM.pdf]

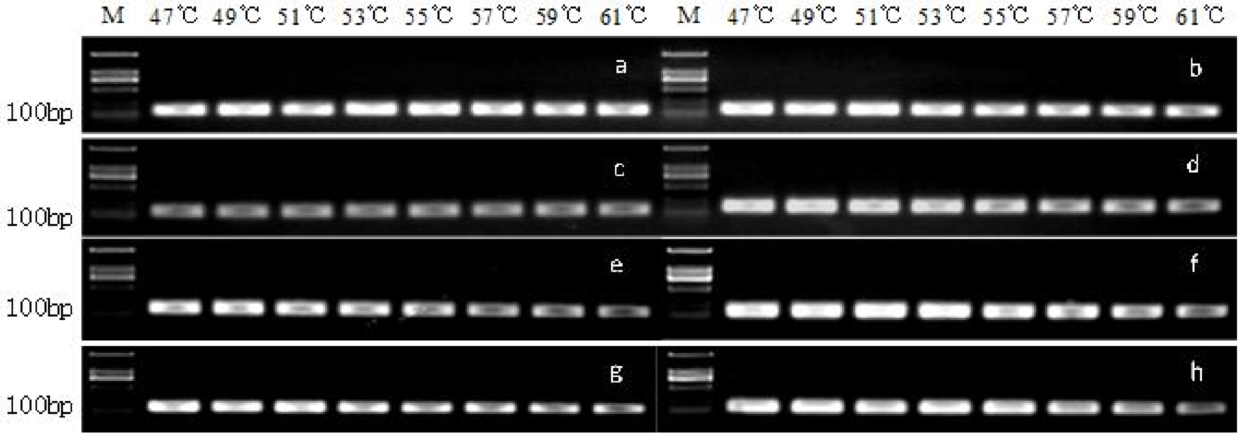

Figure S1. Optimization of annealing temperatures for each component of BBTV. M: DL2000 DNA Marker; a: PCR amplification of DNA- R from 47℃ to 61℃; b: PCR amplification of DNA-U3 from 47℃ to 61℃; c: PCR amplification of DNA-S from 47℃ to 61℃; d: PCR amplification of DNA-M from 47℃ to 61℃; e: PCR amplification of DNA-C from 47℃ to 61℃; f: PCR amplification of DNA-N from 47℃ to 61℃; g: PCR amplification of S2 from 47℃ to 61℃; h: PCR amplification of Sat4 from 47℃ to 61℃.
